# Isolation of Specific Neurons from *C. elegans* Larvae for Gene Expression Profiling

**DOI:** 10.1371/journal.pone.0112102

**Published:** 2014-11-05

**Authors:** W. Clay Spencer, Rebecca McWhirter, Tyne Miller, Pnina Strasbourger, Owen Thompson, LaDeana W. Hillier, Robert H. Waterston, David M. Miller

**Affiliations:** 1 Department of Cell and Developmental Biology, Vanderbilt University, Nashville, Tennessee, United States of America; 2 Program in Neuroscience, Vanderbilt University, Nashville, Tennessee, United States of America; 3 Department of Genome Sciences, University of Washington School of Medicine, Seattle, Washington, United States of America; Inserm U869, France

## Abstract

**Background:**

The simple and well-described structure of the *C. elegans* nervous system offers an unprecedented opportunity to identify the genetic programs that define the connectivity and function of individual neurons and their circuits. A correspondingly precise gene expression map of *C. elegans* neurons would facilitate the application of genetic methods toward this goal. Here we describe a powerful new approach, SeqCeL (RNA-Seq of *C. elegans* cells) for producing gene expression profiles of specific larval *C. elegans* neurons.

**Methods and Results:**

We have exploited available GFP reporter lines for FACS isolation of specific larval *C. elegans* neurons for RNA-Seq analysis. Our analysis showed that diverse classes of neurons are accessible to this approach. To demonstrate the applicability of this strategy to rare neuron types, we generated RNA-Seq profiles of the NSM serotonergic neurons that occur as a single bilateral pair of cells in the *C. elegans* pharynx. These data detected >1,000 NSM enriched transcripts, including the majority of previously known NSM-expressed genes.

**Significance:**

This work offers a simple and robust protocol for expression profiling studies of post-embryonic *C. elegans* neurons and thus provides an important new method for identifying candidate genes for key roles in neuron-specific development and function.

## Introduction

With its well-defined, compact nervous system and facile genetics, *C. elegans* is widely exploited for studies of neural development and function. The morphology and connectivity of the *C. elegans* nervous system is catalogued in comprehensive wiring diagrams [1], [2], [3] that facilitate functional analysis [4], [5]. The unrivaled precision of this nervous system model is complemented by the complete sequence of the *C. elegans* genome and its extensive annotation derived from direct RNA-Seq analysis [6], [7]. Cell-specific profiling experiments have identified subsets of genes that are highly expressed in particular neurons or that may be regulated by transcription factors with key roles in neuron-specific differentiation [8], [9], [10], [11], [12], [13], [14]. Expression profiling substantially narrows the list of candidate genes for tests of function *in vivo* and therefore offers an efficient strategy for identifying critical determinants of neuron differentiation and activity. Fluorescent reporter transgenic lines have been generated for thousands of individual *C. elegans* genes and their expression in specific neurons has been documented [15]. For embryos, cells can be dissociated and neurons that are marked with GFP reporters can be readily isolated by Fluorescence-Activated-Cell-Sorting (FACS) [9], [16], [17]. For example, profiling data generated for BAG sensory neurons by this approach led to the identification of a guanylate cyclase receptor that detects CO_2_ and a conserved ETS transcription factor that regulates BAG neuron fate [18], [19]. For post-embryonic animals, the mRNA-tagging method has been extensively utilized to profile larval and adult neurons [10], [11], [13], [20], [21], [22], [23], [24]. In this strategy, an epitope-tagged mRNA binding protein is selectively expressed in target neurons for immuno-precipitation of cell-specific transcripts [20]. Although useful, this approach requires custom-built transgenic lines and the biochemical preparation may include significant background RNA that limits specificity [21].

The recent development of a simple protocol by Jeff Kuhn’s laboratory for generating dissociated populations of viable cells from *C. elegans* larvae offers the potential alternative to the mRNA tagging method of profiling postembryonic cells isolated by FACS. However, neurons were reportedly under-represented in these preparations [25]. This apparent limitation would restrict ready access to specific types of neurons, the majority of which are rare since they are defined by either a single cell or by bilateral pairs of similar neurons in each animal [1]. But our investigations demonstrate that larval neurons are readily released by the Kuhn cell dissociation protocol. The discrepancy is explained by a requirement in the Kuhn method for rapid adherence of cells to the culture dish. In the first instance, we used FACS to show that GFP-labeled cells comprise at least 30% of viable cells obtained from a transgenic line in which all neurons are marked with a GFP reporter. Specific classes of sensory and motor neurons were also isolated by FACS at a fraction predicted by their relative abundance *in vivo*. To test the applicability of this approach to profiling a specific neuron type, we used FACS to isolate NSM neurons from L1 stage larvae. The NSM neurons consist of two morphologically similar serotonergic neurons located in the pharynx [26], [27]. RNA-Seq profiles confirmed expression of known NSM-specific genes and also detected >1,000 additional transcripts that are enriched in NSM relative to other cell types. We therefore conclude that *C. elegans* larval neurons are readily accessible to isolation by FACS for gene expression profiling and predict that this simple approach will be highly useful for studies of neural development and function in this model organism.

## Results

### Viable *C. elegans* larval neurons can be readily isolated by FACS (Fluorescence Activated Cell Sorting)

**Figure 1:**
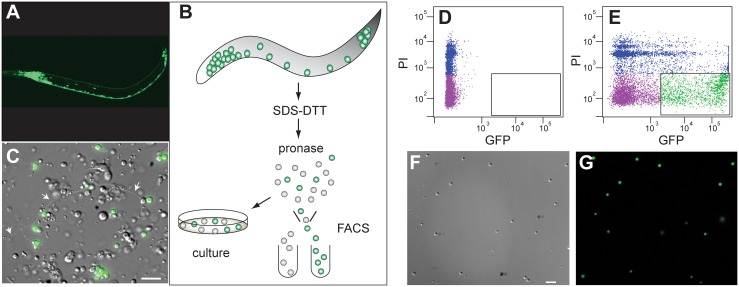
Isolation of *C. elegans* neurons from larval animals by Fluorescence Activated Cell Sorting (FACS). (A) Expression of the pan-neural GFP marker, *evIs111*, in an L2 stage larval animal. Anterior is to left. (B) Procedure for generating dissociated suspensions of larval cells for primary culture and for isolation of GFP-marked cells (green) by FACS. (C) Primary culture of L1-stage cells 36 hr after plating. Arrows point to elongated processes extending from GFP-labeled neurons. Scatterplot of FACS profile for cells dissociated from the wild-type (N2) reference strain (D) and from the pan-neural *evIs111* line (E). Propidium Iodide (PI) marks dead cells (blue). GFP-labeled cells (green) were isolated by FACS (outlined with box). The majority (∼90%) of FACS-derived cells (F) are marked with GFP (G) in primary cultures examined within 2 hr of FACS isolation. Note that neurons are loosely attached which likely accounts for the displacement of DIC vs GFP images of individual cells in these micrographs. Scale bars = 10 micron.

In previous expression profiling studies of embryonic cell-types, we determined that the approximate fraction of a specific cell-type in a FACS (Fluorescence-Activated-Cell-Sorting) profile of all viable cells is correlated with the relative abundance of these target cells in the intact embryo [9], [11], [28]. To test this prediction for larval neurons, we generated primary cultures from L1 larvae labeled with the pan-neural marker, *F25B3.3::GFP* (Fig. 1A, B) [29]. Morphologically distinct, GFP-labeled neurons were well represented in these cultures within 36 hours after plating (Fig. 1C). A FACS profile of the initial dissociated preparation of L1 larval cells detected ∼32% of cells as GFP-labeled (Fig. 1D, E), which compares favorably to the fraction of neurons (222/558 = 40%) in newly hatched L1 larva. Our results stand in contrast to an earlier report that neurons comprise less than 10% of cultured cells derived from L1 larvae. In this previous experiment, the culture medium was replaced within 24 hours of plating and before GFP-labeled cells were counted [25]. We have observed that neurons are only loosely attached at this stage (data not shown) and therefore largely discarded when media are changed after 1 day in culture. In contrast, body muscle cells adhere more quickly, which likely accounts for the previous observation that 81% of cultured L1 cells were derived from muscle, although this fraction comprises only 15% (81/558) of newly-hatched L1 cells [25]. We found that viable neurons from *F25B3.3::GFP* could be isolated by FACS to ∼90% purity by directly counting GFP-labeled cells immediately after sorting (Fig. 1F, G).

**Figure 2:**
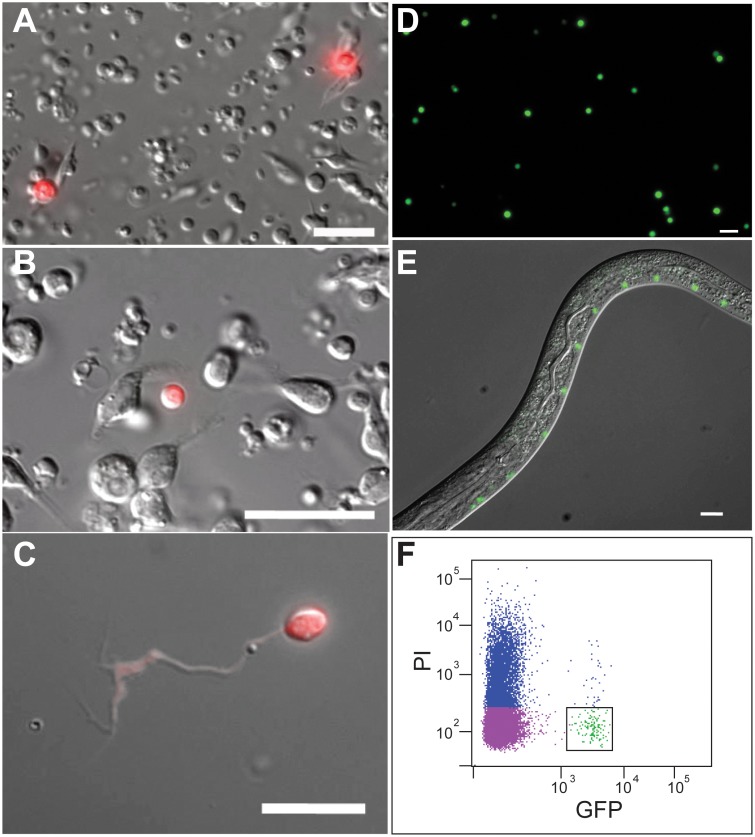
Specific sensory and motor neurons are accessible to isolation by FACS from multiple larval stages. Primary cultures of L1 stage cells 24 hr after dissociating from transgenic lines expressing (A) *mec-4::*mCherry to mark ALM and PLM neurons (red) and (B) *srh-142p::*dsRed to label ADF sensory neurons (red). (C) GABA motor neuron marked with *unc-47::*mCherry (red) and cultured for 24 hour after dissociating from L4 larval animals. (D) Primary culture of A-class motor neurons marked with *unc-4::*GFP (green) and isolated by FACS from L2 stage larvae. (E) *del-1::*GFP labels VB motor neurons (green) in the ventral nerve cord of an L2 stage larva. Anterior to left. (F) FACS profile of cells dissociated from *del-1::*GFP L2 larvae. Propidium Iodide (PI) marks dead cells (blue). Viable GFP-labeled cells (green) are outlined with the box. Scale bars are 10 microns.

We generated dissociated preparations of larval cells labeled with fluorescent markers for sensory and motor neurons to determine if specific neuron types were also accessible to isolation by FACS. Touch neurons (PLM and ALM) and serotonergic sensory neurons (ADF) from L1 larvae (Fig. 2A, B) and GABA motor neurons from L4 stage animals (Fig. 2C) were readily detected in primary cultures. To confirm that specific neuron classes could be purified by FACS, we isolated A-class (Fig. 2D) and VB type motor neurons from L2 stage larvae (Fig. 2E, F). Detection of ADF neurons in culture, which comprise only 0.3% of all L1 cells (2/558), suggested that rare types of neurons could also be isolated by this approach for gene expression profiling.

**Figure 3:**
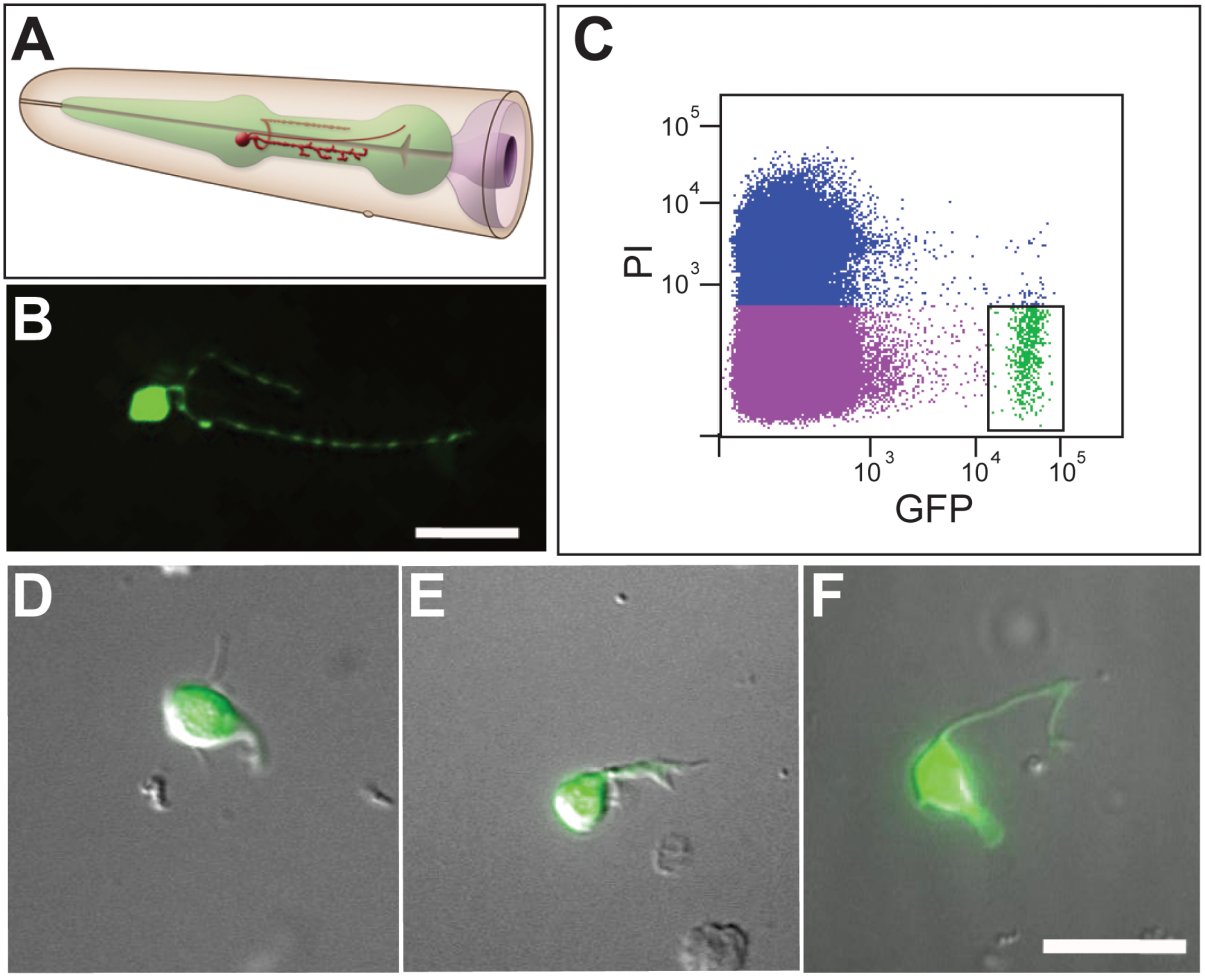
Isolation of NSM serotonergic neurons by FACS. (A) Head region of an adult hermaphrodite depicting the NSML neuron (orange) in the pharynx (green) (WormAtlas). Anterior is to left. (B) Confocal image of NSML in L1 larval stage. Anterior is to left. (C) FACS profile of cells dissociated from L1 stage larvae expressing *tph-1::GFP* in NSM neurons. Propidium Iodide (PI) marks dead cells (blue). Viable GFP-labeled cells (green) are outlined with the box. Images of FACS-isolated NSM neurons (D) 24 hr, (E), 48 hr and (F) 72 hr after plating. Scale bars are 10 microns.

To test the applicability of this strategy to rare neuron classes, we chose the NSM serotonergic neurons for FACS isolation and cell-specific profiling experiments. Two NSM neurons are generated as a left-right pair (NSML, NSMR) in the embryo [30] and function as serotonerigic neurosecretory neurons in the pharynx (Fig. 3A) [31], [32]. To mark these cells, we used a *tph-1::GFP* transgene that exclusively labels NSML and NSMR with a strong GFP signal in L1 larvae [33] (Fig. 3B) (See Experimental Procedures). The lateral branches that are characteristic of adult NSM neurons (Fig. 3A) emerge later in development at the L4 stage [1]. The FACS profile shows that bright *tph-1::*GFP-labeled neurons were readily dissociated from L1 larvae and represented ∼0.3% of all cells, a fraction in good agreement with the *in vivo* proportion (2/558 = 0.36%) of NSM neurons. To confirm viability, FACS-isolated *tph-1::GFP* labeled cells were cultured *in vitro* where they attached to the substrate and initiated process outgrowth (Fig. 3D–F).

### Generating RNA-Seq profiles from small amounts of total RNA

**Figure 4:**
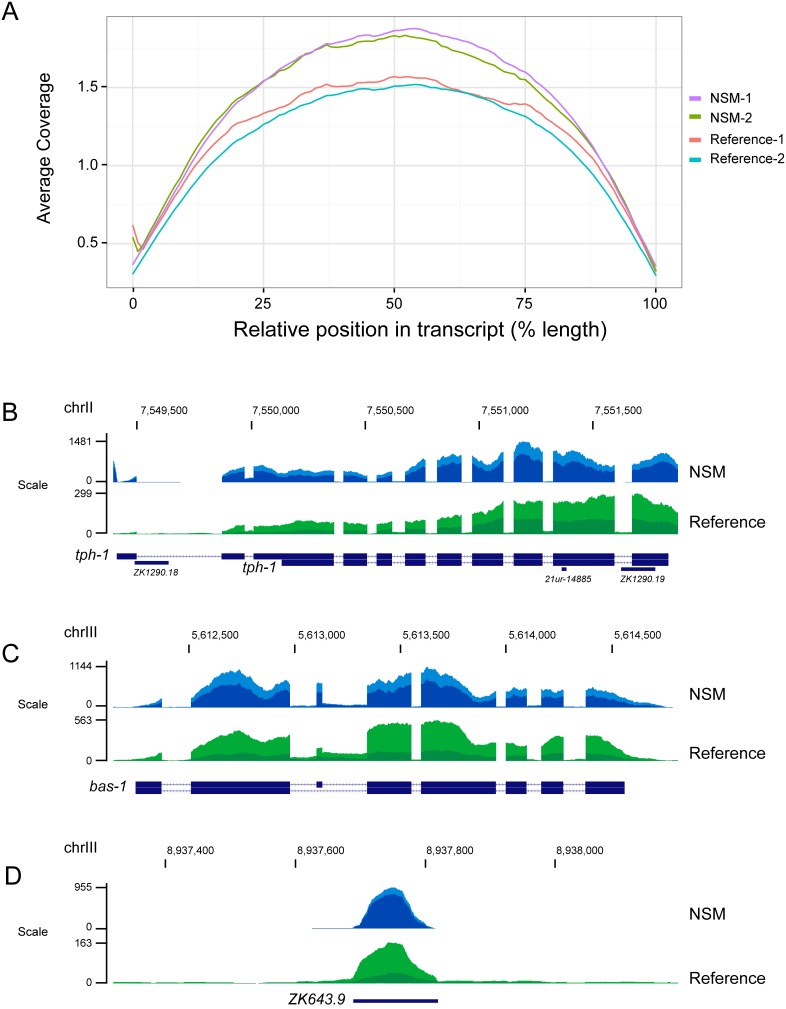
5′-to-3′ coverage. (A) The average relative coverage for each library is shown for normalized transcript length. The Y-axis denotes the average coverage of each position in the transcript normalized to the total number of mapped reads. Coverage for coding sequence genes (B) *tph-1* and (C) *bas-1* and for (D) a small nuclear RNA (snoRNA) gene, *ZK643.9*. Lighter vs darker shades of color depict results for independent NSM (blue) and reference (green) samples.

Preparations of dissociated cells were generated from approximately 3 million L1 larvae. We obtained 5–10 ng of total RNA from 30,000–50,000 FACS-isolated NSM neurons (∼0.25 pg total RNA/cell). Samples were amplified using the NuGEN Ovation V2 protocol to produce cDNA for library construction. Independent replicates were generated from two separate samples of FACS-isolated NSM neurons. Total RNA was also obtained from whole L1 larvae to produce duplicate reference data sets of transcripts expressed in all larval cells at this developmental stage. RNA-Seq data were collected for each sample in a single lane of an Illumina Hi-Seq 2000 to produce >150 million of either PE-75 (paired-end 75) or PE-100 (paired-end 100) reads for each independent library. Each data set yielded at least 8 million reads that map to non-ribosomal RNA genes and detected >10,000 coding genes at a threshold value of ≥1 FPKM (Table S1). This initial list was filtered to exclude transcripts that likely derived from contaminating cells in the FACS-isolated NSM preparation to yield an conservative estimate of 6,200 coding sequence transcripts that are expressed in the NSM neurons (See Experimental Procedures) (File S1). Fig. 4B, C shows average 5′ to 3′ sequence coverage and reads mapping to selected protein coding genes. Fig. 4D features an example of reads mapping to a noncoding RNA gene as expected since the amplification method uses both poly dT and random primers for cDNA synthesis [34].

### RNA-Seq profiles of larval NSM neurons detect highly expressed serotonergic transcripts

**Figure 5:**
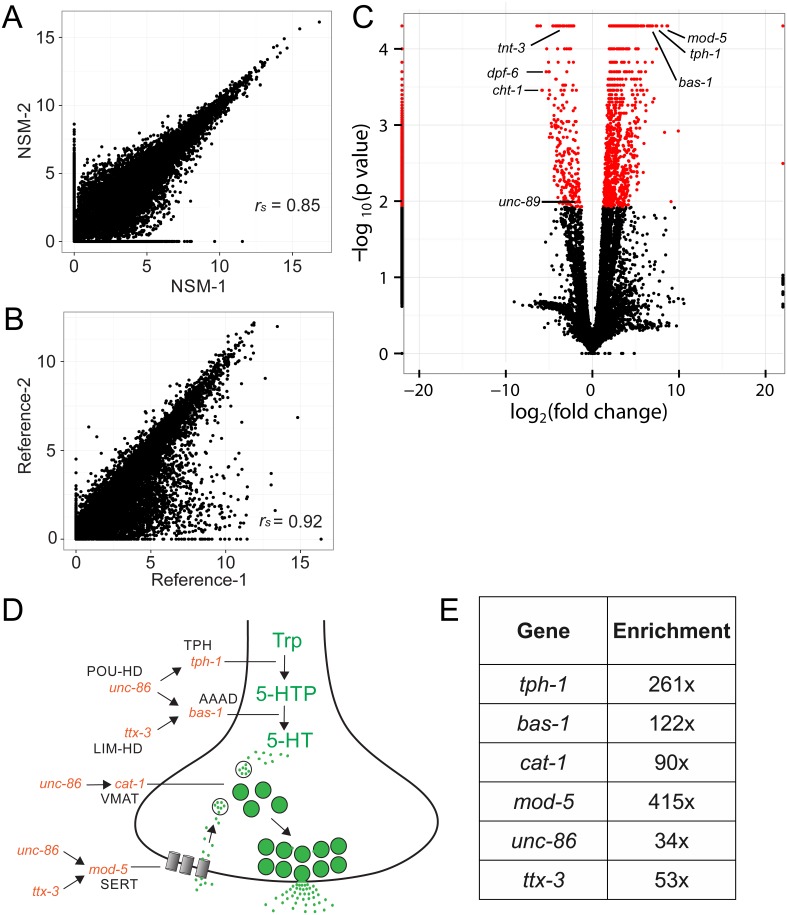
Differential RNA-Seq analysis detects transcripts that are highly expressed in NSM neurons. Pairwise comparisons of (A) NSM and (B) Reference data sets with expression values represented as log_2_(FPKM) and Spearman rank-order correlation coefficients, *r_s_*. (C) Volcano scatter plot of log_10_(p-value) vs log_2_(fold change) of transcript expression in NSM neurons relative to the reference sample derived from all L1 larval cells. Significantly enriched or depleted transcripts (≥2.4 fold, p<0.012) are indicated in red. (D) Schematic of NSM serotonergic presynaptic terminus depicting genes that are highly enriched (E) in the NSM RNA-Seq profile. See text for additional information about specific genes.

**Figure 6:**
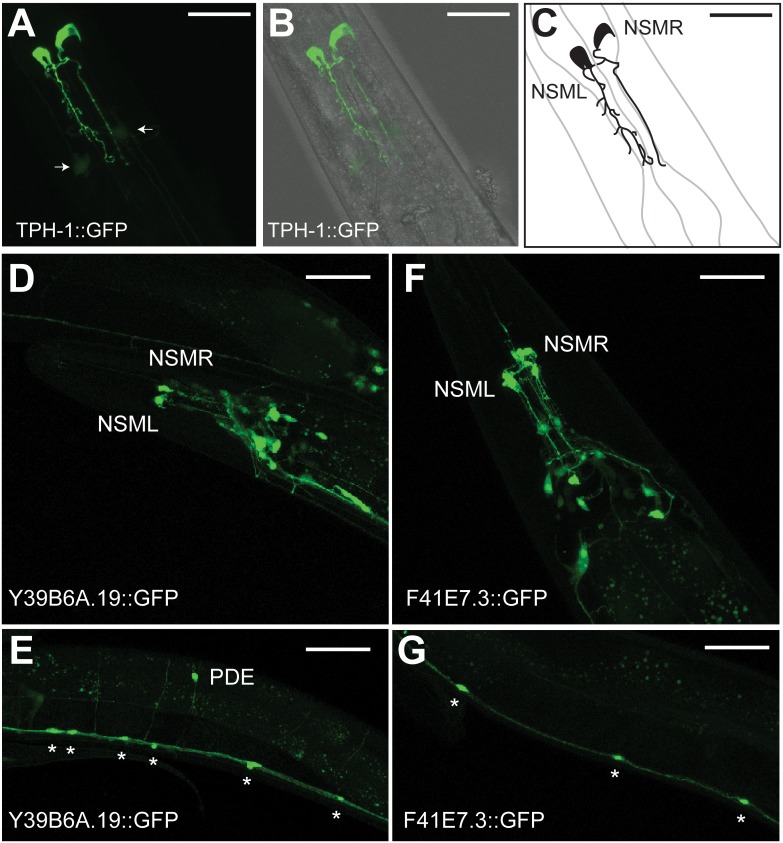
GFP reporters validate NSM-enriched RNA-Seq data set. Transgenic animals expressing promoter::GFP reporter genes for NSM-enriched transcripts. Confocal images of adult hermaphrodites, anterior to left, ventral down. A–B, Bright *tph-1::*GFP (*vsIs45*) expression is limited to NSML and NSMR; note dim *tph-1::*GFP signal in ADF neurons (arrows). C, Cartoon depicting NSM neurons marked with *tph-1*::GFP in the anterior pharyngeal bulb. *Y39B6A.19*::GFP (D, E) and *F41E7.3*::GFP (F, G) are specifically expressed in neurons, including NSM, head neurons and ventral cord motor neurons (asterisks). *Y39B6A.19*::GFP is also expressed in sensory neuron, PDE (E). Scale bar is 25 microns.

Duplicate RNA-Seq data sets for the NSM neurons are well correlated as are the reference samples (Fig. 5A, B) but they show significant differences when compared to each other (*e.g*., NSM vs reference) (Fig. 5C). We detected a total of 1,073 transcripts with significantly elevated expression (≥2.4 fold, ≤0.05 q-value) in the NSM data set *vs* all L1 larval cells (see File S1). To validate this result, we compared the data set of NSM-enriched transcripts to a list of genes annotated in WormBase as previously assigned to NSM by direct observation. The overlap is highly significant with 21 of 36 known NSM genes represented in the NSM-enriched data set (p = 1.6×e^−19^) and an additional 13 genes that are detected at ≥1 FPKM (Table S2). Of particular note in the NSM-enriched list are genes with established roles in NSM differentiation or function. For example, canonical serotonergic genes are highly expressed in NSM. These include enzymes *tph-1* (tryptophan hydroxylase/TPH) and *bas-1* (aromatic amino acid decarboxylase/AAADC) required for serotonin (5-HT) synthesis, a transporter for selective uptake of monamine neurotranmitters into synaptic vesicles, *cat-1* (vesicular monamine transporter/VMAT) and the synaptically localized transporter, *mod-5* (serotonin reuptake transporter/SERT). mRNAs for the POU and LIM homeodomain transcription factors, *unc-86* and *ttx-3*, respectively, which are known to activate expression of these serotonergic genes [31], [35] are also enriched in the NSM profile (Fig. 5C–E). The specificity of this data set is also underscored by the depletion of transcripts that are known to be highly expressed in other tissues or in different developmental periods *e.g*., *tnt-3* and *unc-89* (body muscle), *dpf-6* (pharynx, intestine) and *cht-1* (embryo) (Fig. 5C) [11], [36], [37]. As an additional test of the reliability of the NSM-enriched data set, we scored *in vivo* expression of promoter-GFP reporters for additional genes on this list that have not been previously assigned to NSM. The transcripts selected for this experiment display a broad range of statistical ranks (42–591) and half (2/4) of the corresponding GFP reporters were positively identified as expressed in NSM (Fig. 6) (Table 1). Together, these results strongly support the conclusion that these data sets provide an accurate representation of transcripts that are highly expressed in NSM neurons *in vivo*.

**Table 1:** Expression of promoter-GFP reporters for transcripts enriched in the NSM RNA-Seq data set.

Rank	Gene Name	Protein	Expression	
42	*ZC317.3*	*glc-3*	Glutamate-gated chloride channel	12–14 head neurons, 1 tail neuron
256	*F41E7.3*	*npr-6*	7-TM receptor	NSM, 14–16 head neurons, 5 VNC neurons
297	*Y39B6A.19*	*twk-46*	Tandem pore domain K+ channel	NSM, 15 head neurons, 22 VNC neurons, PDE
591	*F56F3.6*	*ins-17*	Insulin-related peptide	8–10 head neurons, VNC, DNC

Genes are ranked according to statistical significance. VNC (Ventral Nerve Cord), DNC (Dorsal Nerve Cord).

Additional RNA-Seq data sets were generated from these RNA samples after treatment with the DSN (Duplex-Specific Nuclease) method to deplete rRNA sequences [38]. As expected, most of the DSN-treated samples include a higher fraction of reads derived from non-rRNA sequences (Table S1) and show the strongest correlation with the corresponding RNA-Seq data sets obtained from Total RNA (*e.g*., NSM-DSN vs NSM total RNA, Fig. S1). Finally, the total RNA samples were also assayed on tiling arrays [11] (File S3). NSM-enriched transcripts were calculated from the DSN-treated RNA-Seq data sets and from the tiling array results to compile the union of NSM-enriched transcripts (879 genes) showing elevated expression in at least one of the three profiles (*i.e.,* NSM Total RNA, NSM-DSN or NSM tiling array) (See Experimental Procedures) (File S4).

## Discussion

Here we describe a new protocol, SeqCeL (RNA-Seq of *C. elegans* cells), for gene expression profiling of specific larval *C. elegans* cells. Our approach exploits a recently developed method for dissociating larval cells for primary culture [25], [39]. Although this earlier work reported that neurons are poorly preserved in these preparations, we have now demonstrated that diverse neuron types are readily released at fractions predicted by their representation *in vivo*. We showed that GFP-positive neurons constitute approximately 30% of all cells obtained from transgenic L1 stage larval animals expressing a pan-neural reporter; this fraction is comparable to the relative number of neurons in the newly hatched L1 larva (222 neurons/558 total cells; 40%). We have now exploited this finding to demonstrate that viable larval neurons can be readily isolated by FACS for microarray and RNA-Seq analysis. To illustrate the feasibility of this cell isolation procedure for profiling rare neuron types, we produced RNA-Seq profiles for the NSM serotonergic neurons, which occur as a single bilateral pair of cells in the *C. elegans* pharynx [1], [26]. These RNA-Seq data sets identified >6,200 coding sequence transcripts (“Expressed” tab, File S1) in the FACS-isolated cells (See Experimental Procedures). Of these genes, 1,070 unique transcripts are also differentially expressed in the NSM neurons in comparison to all L1 stage larval cells. This NSM-enriched data set was validated by robust elevation of known NSM mRNAs such as genes encoding serotonin-related functions (*e.g., tph-1, bas-1, mod-5*) and the transcription factors that regulate their expression (*e.g., unc-86, ttx-3*) [27], [31]. Thus, these data sets provide a detailed list of NSM-expressed transcripts that are now available for experimental tests to reveal specific roles in NSM development and function.

This new SeqCeL method of using FACS to isolate neurons from *C. elegans* larvae for gene expression profiling complements the previously described application of this approach to embryonic cells (*i.e*., MAPCeL) [8], [9], [14]. Thus, neurons at multiple developmental stages, from embryos to adults (RLM and DMM, unpublished data), are now accessible for RNA-Seq analysis. What are the advantages and disadvantages of this technique in comparison to other approaches? We have previously used the mRNA tagging method, for example, to produce expression profiles of larval neurons. Although this technique is effective, it depends upon the production, in each case, of a custom-built transgenic line in which mRNA can be specifically immuno-precipitated from each target neuron [11], [13], [20], [21]. Gene expression profiles have also been generated from larval cells after microdissection [40] and by methods designed to isolate cell-specific nuclei [41], [42]. To date, however, these approaches have not been applied to neurons. In contrast, the FACS method of cell isolation that we have described here takes advantage of the large number of fluorescent live-cell markers that are already available for most neurons types in *C. elegans* [15] and extensively annotated in WormBase. Furthermore, for neurons that may not be specifically marked with a single reporter gene, pairs of different-colored labels (*e.g*., GFP + mCherry) that are uniquely co-expressed in the target neuron can be readily utilized for FACS isolation [11]; this combinatorial strategy greatly enhances the immediate applicability of our approach [43]. Although we have successfully generated robust neuron-specific profiles, these RNA-seq data sets are dominated by reads derived from ribosomal RNA (rRNA). We have confirmed however, that rRNA sequences can be relatively depleted by the DSN method. Recently, we have also successfully used alternative protocols for excluding rRNA [Ribo-Zero (Invitrogen) and SMARTer (Clontech)] before library construction to produce RNA-Seq data sets from small amounts (<10 ng) of total RNA (RLM, DMM and RHW, unpublished data). These approaches offer the important advantage of reducing the net sequencing bandwidth for each sample. Thus, it should be possible in the future to produce representative neuron-specific profiles of the non-rRNA transcriptome at significantly lower cost. We note the possibility that the cell isolation procedure could potentially alter gene expression. However, our analysis of the NSM data set (Fig. 5D) and of other neuron-specific profiles that we have produced by this method (RLM and DMM, data not shown) has confirmed that known cell-specific transcripts are faithfully represented in these results.

## Conclusion

We have reported a new method, SeqCeL (RNA-Seq of *C. elegans* cells), that uses FACS to purify fluorescently-labeled neurons from *C. elegans* larvae for gene expression profiling by RNA-Seq analysis. We validated this approach by demonstrating that multiple different neuron types are available for FACS isolation and by confirming that an RNA-Seq data set obtained from the NSM serotonergic neurons includes most known NSM-expressed genes. Given the large number and ready availability of neuron-specific markers (*e.g*., GFP, mCherry, etc.) for *C. elegans* it should now be possible to extend this approach a wide array of different neuron classes.

## Experimental Procedures

### Nematode strains

*C. elegans* cultures were maintained as described [42]. N2 was used as the wild-type strain. Additional strains used in this study:

*NW1229 [dpy-20(e1362) IV; evIs111 (F25B3.3::GFP + dpy-20(+))]* [29]
*oyIs51(srh-142p*::dsRed) [44], *LX837 [vsIs45 (tph-1p::GFP)]* [33], *NC197 [dpy-20(e1362) IV*; *wdIs4 (unc-4::GFP + dpy-20(+)]* [45], NC2537 [*unc-119(ed3)*; *wdEx848* (*mec-4::*mCherry *+ unc-119*(+))] [12]; *NC138[dpy-20 (e1282) wdIs3(X)]* [46]; *XE1374 [wpIs39 (unc-47::*mCherry)]
*BC14934 [dpy-5(e907); sEx14934 (rCesZC317.3::GFP + pCeh361)]*
*BC12468 [dpy-5(e907); sEx12468 (rCesF56F3.6::GFP + pCeh361)]*
*BC13337 [dpy-5(e907); sEx13337 (rCesY39B6A.19::GFP + pCeh361)]*
*BC12693 [dpy-5(e907); sEx12693 (rCesF41E7.3::GFP + pCeh361)]*

### Preparation of dissociated larval cells and primary cultures

Worm strains were initially grown on 8P nutrient agar 150 mm plates (see recipe below) seeded with *E. coli* strain NA22 to produce a thick bacterial lawn [21]. For synchronized cultures of L1 larvae, embryos produced by hypochlorite treatment of adult hermaphrodites were allowed to hatch overnight in M9 buffer at 20°C. Approximately 3 million synchronized L1 larvae were typically used for each preparation (Determined by visually counting a sample aliquot on a glass microscope slide). L2 and L4 larval animals were obtained after growth of synchronized L1 larvae on NA22-seeded plates. For isolation of NSM neurons, synchronized L1 larvae were generated from *vsIs45 (tph-1p::GFP)* worms grown on twenty 8P nutrient agar plates (150 mm) seeded with NA22. Bright GFP expression is limited to NSM neurons in *vsIs45* L1 larvae (Fig. 3B, Fig. 6A, B) [33] ADF neurons show dim GFP expression in *vsIs45* but are readily excluded by FACS from the NSM preparation (Fig. 3C). Preparations of dissociated larval cells were generated as previously described [23], [34] with minor changes. L2 and L4 larvae were allowed to settle in cold M9 buffer on ice for 2×30 minute periods to remove bacteria before generating the larval cell prep. Cells were passed through a 5 micron filter at the final step prior to sorting [9]. Primary cell cultures were maintained as previously described [25], [28]. Recipe for 1 liter of 8P nutrient agar: 25 g agar, 20 g bactopeptone, 3 g NaCl [47].

### FACS analysis

Sorting experiments were performed as previously described [7], [9] on a BD FACSAria equipped with a 70 micron diameter nozzle. Propidium iodide or DAPI was included to mark damaged cells. Profiles of GFP and mCherry marker strains were compared to an N2 standard to exclude auto-fluorescent cells. Sorted cells were collected in L-15-10 cell culture medium for primary cultures or in Trizol LS for RNA extraction. Yields of target neurons were calculated as the fraction of potentially available neurons that were isolated by FACS (Table S3). For example, we obtained 30,000–50,000 FACS-isolated NSM neurons from approximately 3 million L1 larvae. With two NSM neurons per animal, 3 million L1 larvae should contain 6 million NSM neurons for an overall yield of <0.85% of purified NSM neurons. FACS yields of <1% were also obtained for other classes of neurons (Table S3). Although yields of sorted neurons are low, this approach is not limiting because large numbers of L1 larvae are easily generated by standard culture conditions (see above).

### RNA extraction and sample preparation for tiling arrays and RNA-Seq analysis

Total RNA was isolated and amplified for application to Affymetrix tiling arrays [9]. RNA integrity and final concentrations were determined in an Agilent Bioanalyzer. For RNA-Seq analysis, purified total RNA was amplified with Ovation RNA-Seq System V2 (NuGEN) and libraries were sequenced using the HiSeq 2000 system (Illumina). The DSN protocol was used to deplete rRNA sequences [38].

### Microarray analysis

A custom chip definition file was generated using probes mapped to gene models for expression quantification as previously described [9]. Tiling array data were quantile normalized and median polished using RMA from the Affy package (v1.43.2) in Bioconductor (v2.14) [48]. A linear model and moderated t-statistic were used to determine differentially expressed genes as implemented by the limma package (v3.21.4) [49].

### RNA sequencing analysis

Sequencing reads were mapped to the *Caenorhabditis elegans* genome and transcriptome (UCSC ce10, Wormbase WS220) using annotation supplied by Illumina (ftp://igenome:G3nom3s4u@ussd-ftp.illumina.com/Caenorhabditis_elegans/UCSC/ce10/Caenorhabditis_elegans_UCSC_ce10.tar.gz). Reads were mapped using the splice-aware aligner tophat2 v2.0.11 [50]. Gene expression quantification and differential expression was analyzed using cufflinks v2.2.1 [51]. FPKM expression values were normalized by upper-quartile normalization [52]; FPKMs were scaled by the ratio of 75^th^ percentile fragment counts to the mean 75^th^ percentile value across all NSM and reference libraries; reads derived from rRNA genes were effectively excluded from this calculation. Gene body coverage analysis was performed using RSeQC v2.3.9. Genomic alignments were visualized using the UCSC genome browser with raw read counts normalized to Reads Per Million mapped reads (RPM). All RNA-Seq data sets used for this work are available at the NCBI Sequence Read Archive (http://www.ncbi.nlm.nih.gov/sra). An independent analysis of these RNA-Seq data sets is described in a separate publication [53].

Hypergeometric probability was calculated with publicly available software at http://www.geneprof.org/GeneProf/tools/hypergeometric.jsp.

Transcripts showing significant expression in NSM neurons were filtered by the test NSM FPKM < reference FPKM to remove false positives that are likely due to contamination from non-GFP cells in the preparation of FACS-isolated NSM neurons [11], [21]. For the union of all three NSM enriched data sets (*i.e.,* NSM Total RNA, NSM-DSN or NSM tiling array), a Bonferroni-style correction was applied to the FDR threshold to limit the accumulation of false positives [54].

### Microscopy

DIC and epifluorescence images of dissociated cells in culture were collected on a Zeiss Axiovert microscope with 40X and 63X objectives. Confocal images were obtained on Leica TCS SP5 and Nikon Eclipse TI confocal microscopes.

## Supporting Information

Figure S1 **Heat-map depicting Spearman correlation coefficients for pair-wise comparisons of RNA-Seq data sets.**
(PDF)

Table S1 **Summary results for RNA-Seq data sets.**
(XLSX)

Table S2 **Known NSM-expressed genes are highly represented in the NSM RNA-Seq data set.**
(DOCX)

Table S3 **Yields for larval neurons isolated by FACS.**
(DOCX)

File S1 **Transcripts detected by RNA-Seq of total RNA from L1 Reference samples and NSM neurons.** File contains worksheets for differential expression results. “All” contains all cuffdiff output. “Significant” contains all genes called differentially expressed ≥2.4X and q-value≤0.05. “Enriched” contains only genes significantly enriched in the NSM neurons versus the reference. “Expressed” lists genes that have a FPKM value >1 in either the NSM samples or reference samples.
(XLSX)

File S2 **Transcripts detected by RNA-Seq of DSN-treated total RNA from L1 Reference sample and NSM neurons.** This file contains worksheets for differential expression results. “All” contains all cuffdiff output. “Significant” contains all genes called differentially expressed ≥3.2X and q-value≤0.05. “Enriched” lists genes significantly enriched in the DSN-treated NSM neuron samples versus the DSN-treated reference. “Expressed” lists genes with FPKM >1 in either the DSN-treated NSM or DSN-treated reference samples.
(XLSX)

File S3 **Transcripts detected by tiling array analysis of total RNA from L1 Reference sample and NSM neurons.** This file contains worksheets for differential expression results of tiling array analysis. “All” contains all limma output. “Enriched” contains only genes significantly enriched in the NSM neurons versus the reference ≥1.5X and ≤5% FDR.
(XLSX)

File S4 **Union of NSM-enriched transcripts from Files S1, S2 and S3.**
(XLSX)
