# Supplementary material for: Isolation of Specific Neurons from C. elegans Larvae for Gene Expression Profiling
Source: PLoS One. 2014 Nov 5;9(11):e112102. doi: 10.1371/journal.pone.0112102 (PMC4221280; doi:10.1371/journal.pone.0112102)
Supplement: Figure S1 — Heat-map depicting Spearman correlation coefficients for pair-wise comparisons of RNA-Seq data sets. (PDF) [file pone.0112102.s001.pdf]

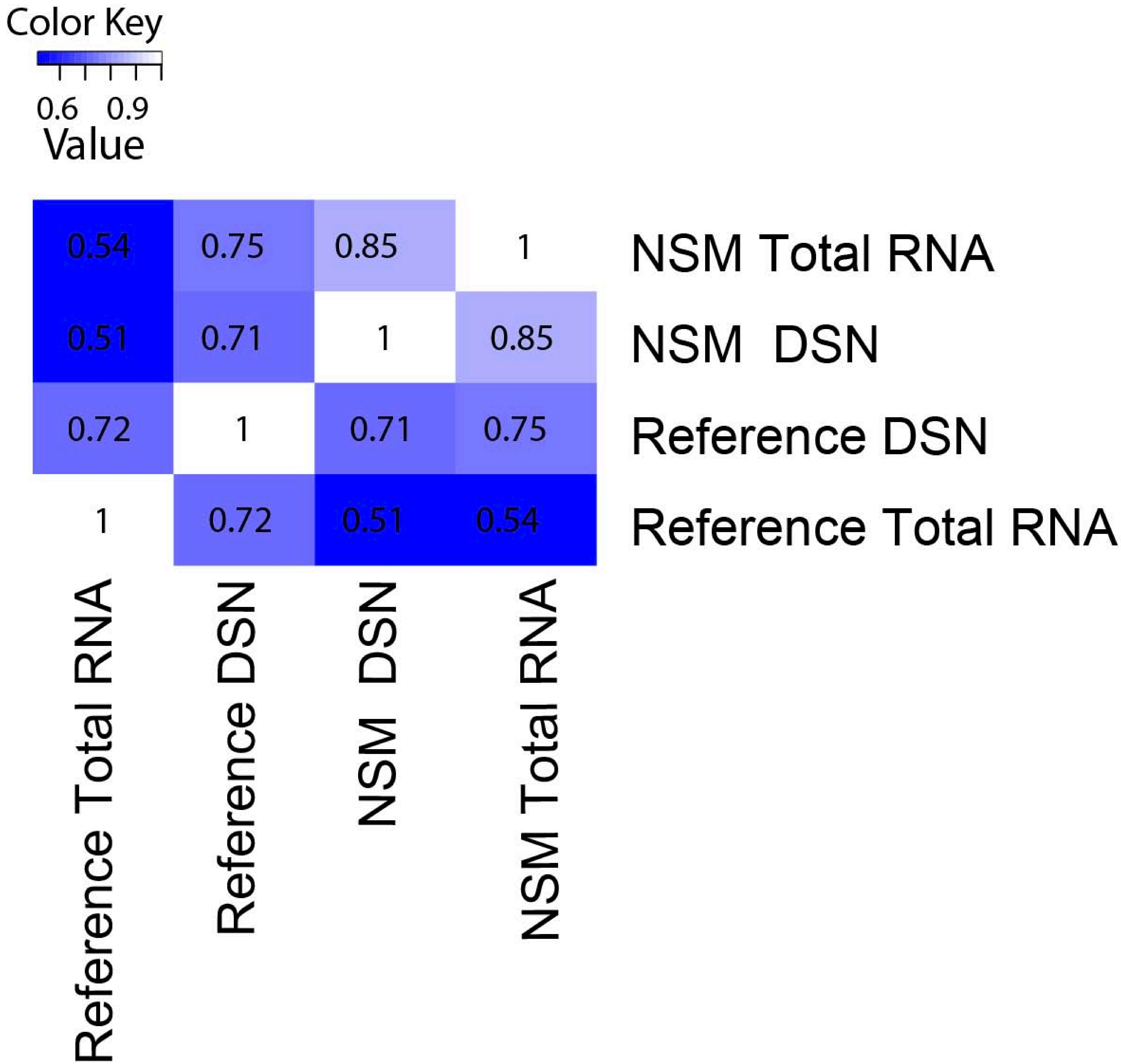
Color Key
0.6
0.9
Value
0.54
0.75
0.85
1
NSM Total RNA
0.51
0.71
1
0.85
NSM DSN
0.72
1
0.71
0.75
Reference DSN
1
0.72
0.51
0.54
Reference Total RNA
Reference Total RNA
Reference DSN
NSM DSN
NSM Total RNA
