# Supplementary material for: Isolation of Specific Neurons from C. elegans Larvae for Gene Expression Profiling
Source: PLoS One. 2014 Nov 5;9(11):e112102. doi: 10.1371/journal.pone.0112102 (PMC4221280; doi:10.1371/journal.pone.0112102)
Supplement: Table S2 — Known NSM-expressed genes are highly represented in the NSM RNA-Seq data set. (DOCX) [file pone.0112102.s003.docx]

**Table S2:** **Known NSM-expressed genes are highly represented in the NSM RNA-Seq data set.** Genes listed in WormBase as expressed in NSM neurons (column 1) are highly represented in the RNA-Seq profile of NSM neurons as either enriched in NSM vs all L1 stage larval cells (Column 2) or detected (> 1 FPKM and > reference) (Column 3).

| **Gene** | **Enriched in NSM** | **Detected in NSM** |
| --- | --- | --- |
| *aho-3* |  |  |
| *bas-1* | X | X |
| *cat-1* | X | X |
| *cat-4* |  | X |
| *ceh-2* | X | X |
| *dkf-1* |  |  |
| *dkf-2* |  | X |
| *eat-4* |  | X |
| *flp-4* |  | X |
| *flp-2* |  | X |
| *glr-8* | X | X |
| *goa-1* | X | X |
| *hst-3.1* | X | X |
| *ins-1* | X | X |
| *mgl-1* | X | X |
| *mgl-3* | X | X |
| *mig-10* | X | X |
| *mod-5* | X | X |
| *mpz-1* | X | X |
| *ncam-1* |  |  |
| *nlp-13* | X | X |
| *nlp-18* |  |  |
| *nlp-19* |  | X |
| *ntc-1* |  |  |
| *rig-3* | X | X |
| *rig-6* | X | X |
| *ser-2* |  | X |
| *ser-4* |  | X |
| *spp-12* |  |  |
| *srab-4* | X | X |
| *tbx-2* |  |  |
| *tph-1* | X | X |
| *tyra-2* | X | X |
| *unc-103* | X | X |
| *unc-86* | X | X |
| *unc-9* | X | X |
