# Supplementary material for: Isolation of Specific Neurons from C. elegans Larvae for Gene Expression Profiling
Source: PLoS One. 2014 Nov 5;9(11):e112102. doi: 10.1371/journal.pone.0112102 (PMC4221280; doi:10.1371/journal.pone.0112102)
Supplement: Table S3 — Yields for larval neurons isolated by FACS. (DOCX) [file pone.0112102.s004.docx]

**Table S3. Yields for larval neurons isolated by FACS.**

| **Marker** | **Larval Stage** | **Neuron** | **# of Neurons/ larva** | **Cells isolated by FACS**  **x 10^3^** | **Viabilty^1^** | **Yield^2^** |
| --- | --- | --- | --- | --- | --- | --- |
| *F25B3.3::GFP* | L1 | All neurons | 222 | 200 - 700 | 45% | <0.1%^3^ |
| *tph-1::*GFP | L1 | NSM | 2 | 30 - 50 | 65% | <0.85% |
| *unc-4::*GFP | L2 | I5, SAB, DA | 13 | 50 - 150 | 80% | <0.4% |
| *del-1::*GFP | L2 | SABV, VB | 13 | 10 - 40 | 51% | <0.1% |
| *unc-47::*mCherry | L4 | DD, VD, RME, AVL, RIS, DVB | 26 | 100 - 150 | 93% | <0.2% |

^1^Viabilty was calculated as the average fraction of cells that did not stain with either propidium Iodide or DAPI in FACS profiles for the indicated neuron.

^2^Yields are calculated for the fraction of available neurons that were isolated by FACS from 3 million larval animals. For example, *vsIs45 (tph-1p::GFP*) is expressed in 2 NSM neurons/L1 stage larva. Thus, the maximum observed yield for FACS-isolated NSM neurons = 50,000/6 million = 0.83 %.

^3^Underestimates the actual yield because FACS sessions were stopped early (30 - 60 min) before the entire sample was sorted due to the high number of isolated neurons.
